# Remote patient monitoring after cardiac surgery: The utility of a novel telemedicine system

**DOI:** 10.1111/jocs.15962

**Published:** 2021-09-03

**Authors:** Kıvanç Atilgan, Burak E. Onuk, Pınar Köksal Coşkun, Fahri G. Yeşi̇l, Cemal Aslan, Abdullah Çolak, Aksüyek S. Çelebi̇, Hüseyin Bozbaş

**Affiliations:** ^1^ Department of Cardiovascular Surgery TOBB ETU Hospital Ankara Turkey; ^2^ Department of Cardiology TOBB ETU Hospital Ankara Turkey

**Keywords:** cardiac surgery, real‐time ECG, remote Holter ECG, remote patient monitoring, telemedicine

## Abstract

**Objective:**

We examined cardiac surgery patients who underwent monitoring of postoperative vital parameters using medical monitoring devices which transferred data to a mobile application and a web‐based software.

**Methods:**

From November 2017 to November 2020, a total of 2340 patients were enrolled in the remote patient monitoring system after undergoing cardiac surgery. The medical devices recorded vital parameters, such as blood pressure, pulse rate, saturation, body temperature, blood glucose, and electrocardiography were measured via the Health Monitor DakikApp and Holter ECG DakikApp devices which reported data to web‐based software and a mobile application (DakikApp Mobile Systems, Remscheid, Germany). During the follow‐up period, patients were contacted daily through text and voice messages, and video conferences. Remote Medical Evaluations (RMEs) concerning patients' medical states were performed. Medication reminders, daily treatment were communicated to the patients with the DakikApp Mobile Systems Software.

**Results:**

During a mean follow‐up period of 78.9 ± 107.1 (10–395) days, a total of 135,786 patient contacts were recorded (782 video conferences, 2805 voice messaging, and 132,199 text correspondence). The number of RMEs handled by the Telemedicine Team was 79,560. A total of 105,335 vital parameter measurements were performed and 5024 hospital application requests (6.3% per RME) were addressed successfully and hospitalization was avoided. A total of 144 (6.1%) potentially life‐threatening complications were found to have been diagnosed early using the Telemedicine System.

**Conclusion:**

Remote Patient Monitoring Systems combined with professional medical devices are feasible, effective, and safe for the purpose of improving postoperative outcomes.

## 1 INTRODUCTION

Technological innovations and advances have reflected strongly upon the medical field, especially in recent years. These developments have had a significant impact on life expectancy, which has resulted in an increase in life expectancy, and thus, a numerical increase is observed in the number of patients who require cardiac surgery. Older patients with high comorbidity are now undergoing cardiac surgery much more frequently, and the difficulty of caring for such patients is increasing with every passing day.^1^, ^2^, ^3^, ^4^ Cardiac surgeons can reduce pre‐ and postoperative mortality rates by using innovative techniques, as well as by keeping up with the pace of developments during and after treatment.^5^, ^6^, ^7^ Similar to the effects of advanced surgical techniques which have reduced morbidity and mortality in cardiac surgery,^8^, ^9^ technology‐assisted approaches to remote patient follow‐up have the potential to revolutionize postoperative care–particularly during and after the coronavirus disease 2019 (COVID‐19) pandemic.

Follow‐up after discharge is undoubtedly a primary part of care in Cardiac Surgery, and it has become a critical point due to the increasing age and comorbidities of patients. At the same time, the accessibility of knowledge via the internet has triggered an increase in the enthusiasm of patients who try and understand their illness by accessing available data. Smartphones, which have features similar to computers and are carried by almost everyone, have been demonstrated to have potential as medical monitoring systems for patients.^10^, ^11^, ^12^

Although heart diseases are generally chronic, the management of patients progresses rapidly in the presence of surgery indication, and the surgery itself is usually performed as early as possible and hospitalization is quite short. As a result, patients are usually discharged to their homes while they still carry the psychological “shock” of the surgery. In the first month after discharge, delayed bleeding at the surgical site, wound infection, pleural or pericardial fluid formation, arrhythmias, and so forth may develop as serious complications. Additionally, irregular drug use, ineffective mobilization, disruptions in treatment and control processes, postoperative adaptation difficulties such as presenting high anxiety for normal postoperative progress, especially in postoperative pain management, and rehospitalization can also leave patients in difficult situations. To address these problems, some developed countries have structured routine rehabilitation processes in facilities that provide care to patients after cardiac surgery; however, others do not have such infrastructure.^13^, ^14^, ^15^ The lack of adequate follow‐up after major surgeries may be an important problem due to the aforementioned risks, and therefore, the possibility and benefits of conducting remote patient follow‐up must be explored, especially considering the impact of COVID‐19 on the method and application of patient management.

In this study, our aim was to assess the results of utilizing a sophisticated telemedicine solution for postoperative follow‐up in patients discharged after cardiac surgery.

## 2 MATERIAL AND METHODS

Between November 2017 and November 2020, a total of 2340 patients who underwent cardiac surgery at the Department of Cardiac Surgery of TOBB ETU University were enrolled for postoperative follow‐up with a Remote Patient Monitoring System (DakikApp Mobile Systems, Remscheid, Germany). A Telemedicine Department was established to run this follow‐up system, and six doctors and 15 nurses, who had been educated previously in clinical interactive educations, were assigned to the department. Medical consent forms were obtained from all patients for the use of Telemedicine follow‐up and all clinical data were stored in accordance with the Law on Protection of Personal Data.

Patients' demographic characteristics, ejection fraction (EF) values, body mass index (BMI, weight [kg] divided by height [m] squared), preoperative EURO II risk scores (as expected mortality percentage, %), comorbidities, diagnoses, and applied procedure(s) and procedural properties (combined procedure and redo procedure) were recorded. During postoperative evaluations, causes for rehospitalization and the number of hospital admissions prevented with the utilization of remote patient management were recorded.

### 2.1 Remote monitoring system

**Figure 1:**
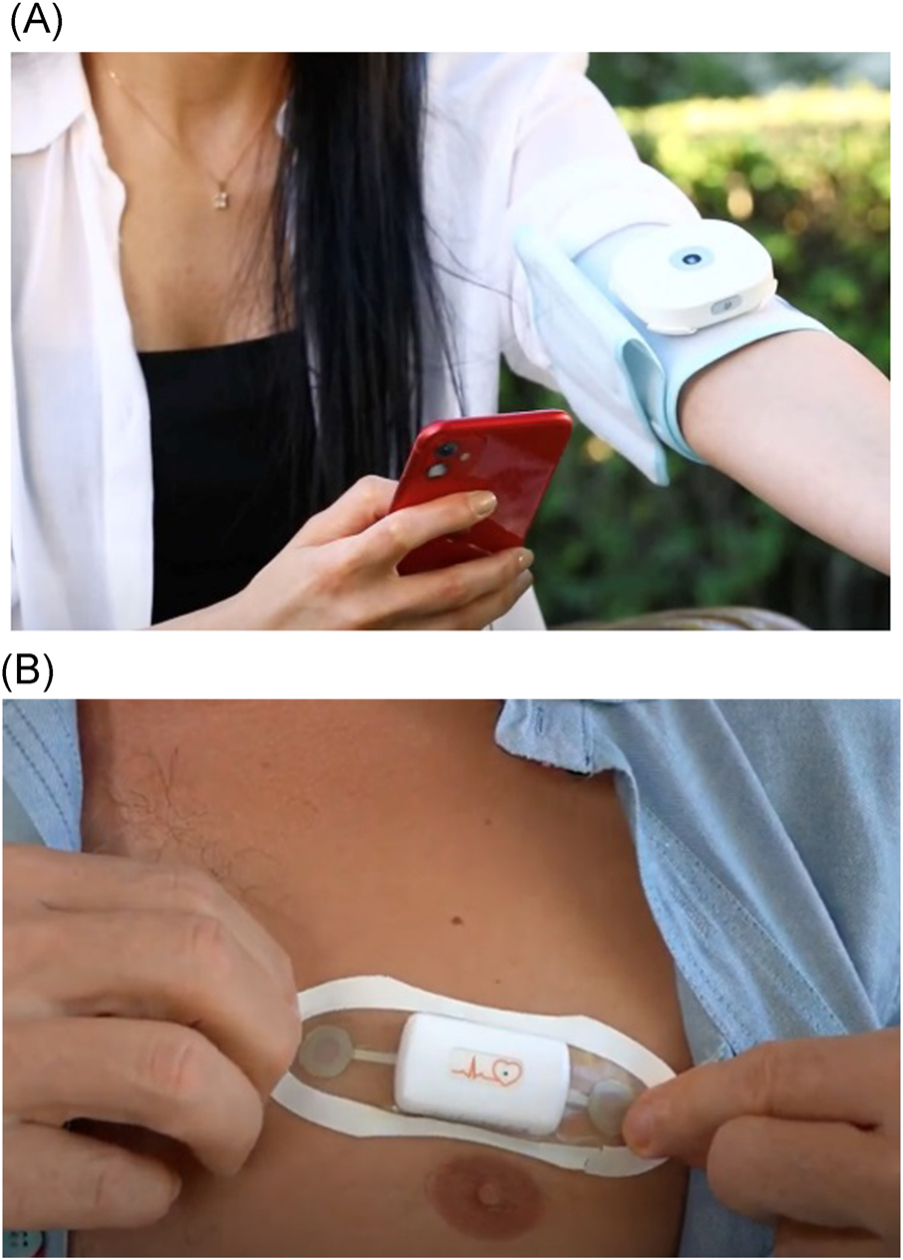
Medical devices and technical specifications used for Remote Patient Monitoring (A) Health Monitor DakikApp: blood pressure, heart rate, O_2_‐saturation, blood glucose; (B) Holter ECG DakikApp: 24 h Holter ECG (One Channel), live ECG, heart rate variability, telemetry)

**Figure 2:**
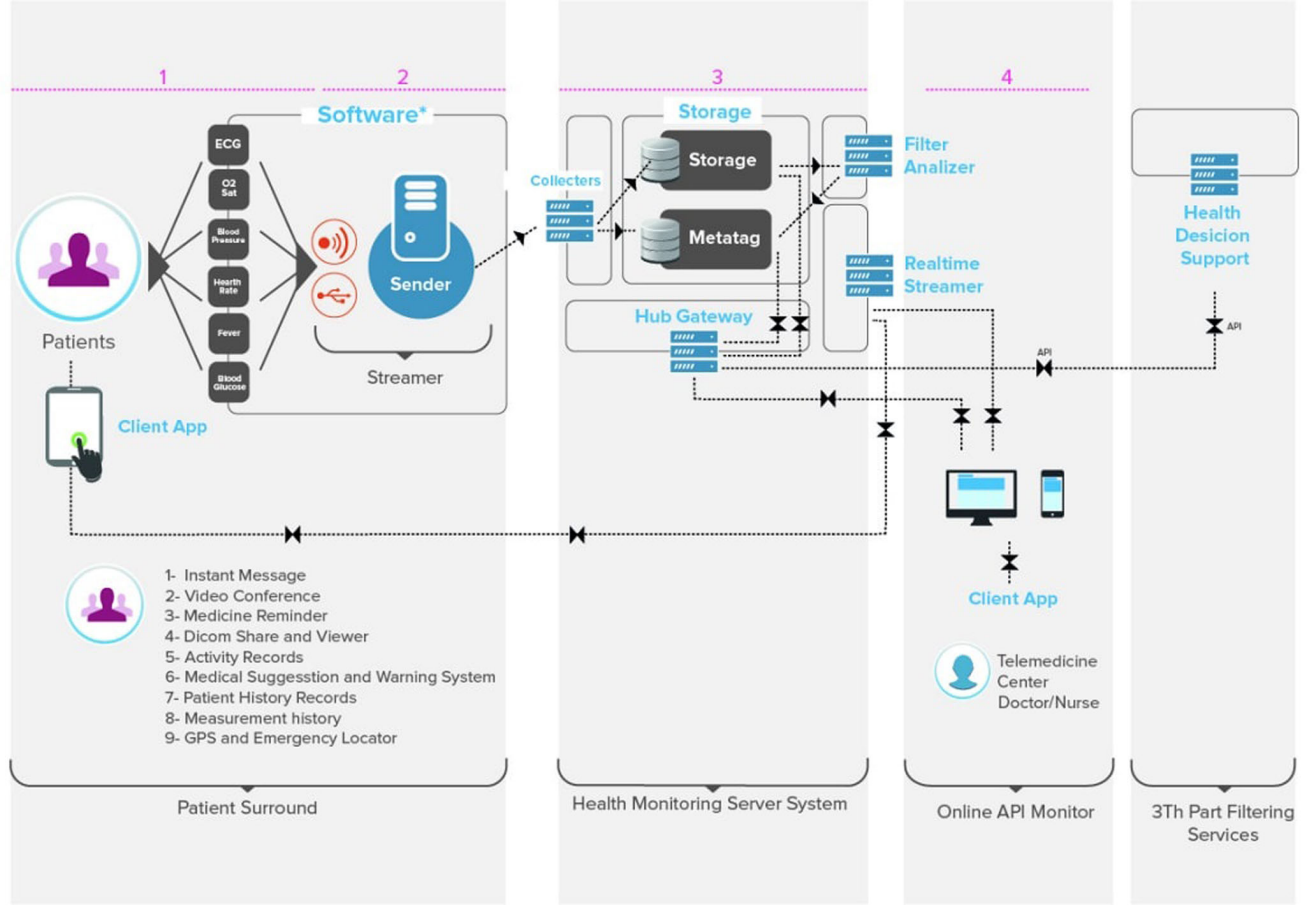
Infrastructure of the Remote Patient Software System and both visual data transfer and system features

The patients had been hospitalized for 5.8 ± 2.8 days after heart surgery, and they underwent training for the use of the DakikApp Mobile Systems system before discharge. The system consisted of three major components: the mobile application (installed on each patient's smartphone), the DakikApp health monitor device (which measures six vital parameters: blood pressure, heart rate, oxygen saturation, body temperature, blood glucose, and electrocardiography [ECG]), and the Holter ECG DakikApp device (provides single‐channel live ECG monitoring) (Figure 1). The system also includes medication reminder, suggested daily life activities, diet and nutrition plans, and a video conference and communication platform (Figure 2). Patients who had been prescribed warfarin medication (*n* = 992, 42,3%) were given a CoaguChek device for the measurement of International Normalized Ratio (INR) at home. By means of interactive communication ability with a clinician, they were able to adjust their warfarin doses without the need for visiting any healthcare institution.

All vital parameters measured by the patients at their homes were instantly transferred to the Telemedicine team (Remote Medical Evaluation [RME]). Every day, each patient was clinically evaluated via the RemotePatient Monitoring System (DakikApp Mobile Systems Remote Patient MonitoringSystem, Remscheid, Germany) and RMEs were repeated within the same day if deemed necessary. The patients' health states were also checked through virtual visits with the use of the communication platform, when needed. Any and all patient‐specific pathological data was immediately put to the attention of the related clinician through a notification system. According to a pre‐determined Event Protocol (Table 4) for possible complications and events, an electronic Event Form was filled out for each patient. After the completion of planned follow‐up duration for each patient, remote monitoring was continued for up to 1 year when necessary.

Informative articles associated with the specific diagnosis and treatment of the patient were prepared and uploaded into the software system (Warning/Suggestion Module), and these articles were automatically sent to patients through an algorithm so that the patients could receive daily information about their condition. Thus, while the patients were informed about their own disease(s), they were also able to obtain relevant information, including the benefits or side effects of the drugs they used, what their considerations should be during the postoperative period, how to adhere to a healthy and effective diet, and the activities that they could partake in during the treatment period.

### 2.2 Clinical outcomes of remote monitoring

Medical care events associated with remote follow‐up data or RMEs were classified as follows: preventing erroneous medication use, preventing dosage errors, adjustment of medication(s) according to findings, wound care monitoring, constipation/diarrhea treatment, sleep problems treatment, pain management, warfarin dose adjustment, remote home care training, nutrition, and fluid consumption assessment and recommendations. Causes for rehospitalization and complaints/conditions associated with prevented repeat admissions were also recorded.

Patients were able to express their request to apply to the hospital through the application and these requests were recorded by the Telemedicine team. After meticulous evaluation of data and reported symptoms, patients deemed to have very low risk were remotely advised about their concerns and received remote medical care or treatment according to their condition. All individuals were informed in no uncertain terms that the final decision to apply to the hospital was theirs if their concerns continued or remote medical care was insufficient in ameliorating their complaints.

### 2.3 In‐person follow‐up

Patients were scheduled for follow‐up visits at the first, third and twelveth postoperative months. Their general condition was examined by physical examination, wound control was performed, and chest X‐ray, echocardiography, ECG, and necessary blood tests were ordered. Apart from these controls, the patients were called to our clinic or the nearest health center when deemed necessary, and relevant clinical examinations causing in‐person visits were performed and recorded. In this way, the findings detected during remote monitoring were re‐examined under hospital conditions and the results were matched.

### 2.4 Data collection and analysis

All data from IOS, Android, and web‐based systems were collected in the SQL and NO‐SQL 8.0 databases. Login was performed with a two‐factor authentication security system, and all patient data was stored with 256‐bit encryption. Patients' demographic data were inserted into the live statistics panel, called Grafana Labs (Version 6.7), which provided continuous data‐flow display, and thus, deviations or incorrect entries were evaluated instantly and corrected. The GraphPad Prism version 9.0 software was used to obtain descriptive statistics and graphical representations of the demographic characteristics of patients and the data recorded with the use of the remote patient monitoring system and in‐person assessments, including all vital parameters and RME‐related results. Data and frequencies were described relative to the number of RMEs, when applicable.

## 3 RESULTS

A total of 2340 patients were included in the remote monitoring program after undergoing cardiac surgery. The mean age of the patients was 61.2 ± 13.1 (20–89) years and the mean preoperative Euro Risk II score was 5.3 ± 5.1% (0%–22%). The summary of patient characteristics is depicted in Table 1. Isolated coronary artery bypass grafting was applied in 1192 (51%) patients, valve replacement in 514 (22%) patients, and combined procedures were applied in 842 (36%) patients.

**Table 1:** Patient demographics, surgical, and medical characteristics

	Patients (*n* = 2340)
Demographics	
Mean age (year)	61.2 ± 13.1 (20–89)
Sex (M/F)	1356 (58%)/984 (42%)
LVEF (%)	51.2 ± 10.6 (20–70)
Preoperativ Euro Risk (%)	5.3 ± 5.1 (0–22)
Body Mass Index (kg/m^2^)	1.94 ± 0.17 (1.56–2.4)
Surgical characteristics	
Isolated CABGs	1192 (51%)
Valve replacements	514 (22%)
Valve repairs	422 (18%)
Aortic surgeries	140 (6%)
Endovascular EVAR/TEVAR	72 (3%)
Combined procedures	842 (36%)
Re‐do procedures	374 (16%)
Medical characteristics	
Hypertension	1380 (59%)
Diabetes	748 (32%)
Chronic renal failure	398 (17%)
Dialysis	72 (3%)
COPD	561 (24%)
Emergencies	202 (8,6%)
Length of postop. Hospital stay (day)	5.8 ± 2.8

Abbreviations: CABG, coronary artery bypass grafting; COPD, chronic obstructive pulmonary disease; EVAR, endovascular aortic repair; LVEF, left ventricle ejection fraction; TEVAR, thoracic endovascular aortic repair.

The patients included in the remote monitoring program were hospitalized for an average of 5.8 ± 2.8 days. Among the 2340 patients, 12 (0.5%) lost their lives during the follow‐up. Eight (0.3%) patients died due to noncardiac etiologies (renal failure [4], COVID‐19 [2], stroke [2]) and 4 (0.2%) patients due to cardiac causes. The remote monitoring of 2328 patients was completed as planned. The mean follow‐up period was calculated as 78.9 ± 107.1 (10–395) days overall. A total of 79,560 RMEs (0.92 ± 0.2 per patient/day) had been performed. The number of total vital parameter measurements made in patients during the follow‐up period and the patient averages for each parameter are presented in Table 2.

**Table 2:** Results (values are presented as mean ± *SD* and *n* (%))

	Patients (*n* = 2340)	
Remote monitoring time (days)	78.9 ± 107.1 (10–395)	
Communications		(per patient/day)
Text messages	132.199	1.53 ± 0.22
Voice messages	2.805	0.12 ± 0.04
Video conferences	782	0.03 ± 0.01
Remote Medical Evaluation of Telemedicine Team (RME)	79.560	0.92 ± 0.24
Number of measurements		(per patient/day)
Blood pressure	22.282	0.12 ± 0.01
Heart rate	23.617	0.13 ± 0.01
Blood oxygen saturation	23.617	0.13 ± 0.01
Body temperature	21.338	0.11 ± 0.01
Blood sugar	8.992	0.04 ± 0.01
Holter/event ECG (Live)	5.489	0.03 ± 0.01
Total	105.335	0.57 ± 0.05
Medical care events		
Avoiding wrong medication use		
Avoiding wrong doses of medication		(% per RME)
Adjusting medication according to the	628	0.7
Findings	422	0.5
Wound care monitoring	3.377	4.2
Constipation/diarrhea treatment	2.108	2.6
Sleep problems treatment	311	0.3
Pain management	2.124	2.6
Warfarin dosage adjustment*	1.834	2.3
Remote home care process training	8.880	11.1
Nutritional fluid consumption	2.820	3.5
Recommendations	3.056	3.8
*n* (total)	25.560	32.1
Early recognition of complications**		(% patient)
Pericardial effusion	118	5.0
Pleural effusion	167	7.1
Supraventricular tachycardia	143	6.1
Cardiac ischemia	8	0.3
Renal failure	35	1.4
Cardiac decompensation	18	0.7
INR value disorder	79	3.3
COVID‐19 infection	44	1.8
*n* (total)	612	26.1

Abbreviations: ECG, electrocardiogram; INR, International Normalized Ratio (for prothrombin time); RME, Remote Medical Evaluation of Telemedicine Team; *SD*, standard deviation.

* Only warfarin users (*n* = 992).

** With or without re‐hospitalizations for each patient.

The patients used the system to communicate with the Telemedicine Team a total of 135,786 times. Detailed descriptions are as follows: text messages 132,199 times (1.53 ± 0.22 per patient/day), voice messages 2805 times (0.12 ± 0.04 per patient/day), video conferences 782 times (0.03 ± 0.01 per patient/day) for remote follow‐up and remote postoperative recommendations for patients who underwent heart surgery, sternum, and extremity surgical wound care. The total number of medical care events experienced in patients followed up after RME was determined to be 25,560 (32.1% per RME). The most common medical care event was adjustment of warfarin dose adjustment, performed a total of 8880 times (11.1% per RME) (Table 2).

With the remote monitoring system, mild, and potential life‐threatening complications were diagnosed in a total of 612 (26.1%) cases in the early period, and, as a result, 144 patients (6.1%) were hospitalized and underwent relevant treatments (Table 3). Patients who had various complications but were not re‐hospitalized (*n* = 468, 20%) were treated remotely by altering drug dosage(s) or providing various suggestions via video conference. Severe pericardial effusion was detected in 19 (0.8%) patients, heart rhythm disorders in 33 (1.4%) patients, and left ventricular decompensation in 18 (0.7%) patients. Among a total of 44 (1.8%) individuals diagnosed with COVID‐19 during follow‐up, five (0.2%) had been admitted for treatment. Six (0.2%) patients were treated with severe INR disorder (INR > 7) in the hospital for one day. The Telemedicine team determined that 118 (82%) of 144 patients who were re‐hospitalized had conditions that were severe enough to cause serious hemodynamic disorder and life‐threatening complications if the diagnosis had been delayed in comparison to current literature (See Event Protocol, Table 4). Of the 144 patients hospitalized, 92 (64%) received interventional treatment (19 [13%] pericardial drainage, 24 [16%] pleural puncture, 20 [14%] cardioversion, 8 [0.5%] coronary angiogram, 5 [3.4%] dialysis, 10 [7%] wound revision, and 6 [4.1%] sternal revision). Twenty (14%) of the remaining 52 (36%) patients received temporary intensive care treatment. Of note, all patients who had died (*n* = 12) had been called for hospitalization according to RME findings and remote communication outcomes.

**Table 3:** Rehospitalization (values are presented as mean ± *SD* and *n* (%))

Rehospitalization	Patients (*n* = 2340)	% (per patient)	Event protocol
Necessary rehospitalization			
Severe pericardial effusion	19	0.8	1A
Severe pleural effusion	24	1.0	2A, 2B
Heart rhythm disorders	33	1.4	3A, 3B, 3C
Cardiac ischemia	8	0.3	4
Stroke	5	0.2	5
Renal failure	10	0.4	6
Cardiac decompensation	18	0.7	7
INR value disorder	6	0.2	15B
COVID‐19 infection	5	0.2	8
Sternal/wound healing problem	16	0.6	9A
*n* (total)	144	6.1	
Prevention of unnecessary repeat admissions*			
Severe pain		**% (per RME)**	
Hypertensive attack	2228	2.8	10
Low blood pressure	687	0.9	11A
Severe dyspnea	294	0.4	11B
Asymptomatic pericardial and pleural	576	0.7	12
Effusions	242	0.3	1B
Secretion at the wound site	191	0.2	9B
Lower extremity edema	14,822	0.2	13
Allergic reaction	332	0.02	14
Sinus tachycardia	198	0.4	3B
Atrial fibrillation	106	0.2	3A
INR value disorder		0.1	15A
*n* (total)	5024	6.3	

Abbreviations: COVID‐19, coronavirus disease 2019; INR, International Normalized Ratio (for prothrombin time); RME, Remote Medical Evaluation of Telemedicine Team; *SD*, standard deviation.

* More than one medical event in the same patient was included in the calculation.

**Table 4:** Description of potential medical complications and their indications for treatments

No.	Event protocol title	Event protocol
1	Pericardial effusion	
1A	Severe	Symptomatic and intervention needed (>3 cm at diastole, circular)
1B	Asymptomatic	Asymptomatic cases, responsive to anti‐inflammatory medical therapy
2	Pleural effusion	
2A	Severe	Symptomatic and intervention needed (filling 1/3 of pleural cavity)
2B	Asymptomatic	Asymptomatic cases, responsive to anti‐inflammatory, and diuretic therapy
3	Heart rhythm disorders	
3A	Atrial fibrillation	Symptomatic tachyarrhythmia absolute with hypotension and clinical worsening
3B	Sinus tachycardia	Symptomatic or asymptomatic cases, responsive to medical therapy
3C	Ventricular extrasystole	Symptomatic or asymptomatic cases, responsive to medical therapy
4	Cardiac ischemia	Clinically symptomatic ischemia proven by ECG findings requiring intervention
5	Stroke	Clinically proven cerebrovascular attack requiring intervention
6	Renal failure	Anuria or oliguria requiring hospitalization. condition requiring dialysis
7	Cardiac decompensation	Left ventricular failure, need for inotropic drugs, intensive care treatment
8	COVID‐19 infection	Symptomatic COVID‐19 infection, viral pneumonia
9	Sternum/wound	
9A	Healing problem	Sternal instability and severe wound infection requiring intervention
9B	Secretion at the wound site	Wound discharge and partial healing impairment
10	Severe pain	A desire to go to the hospital for severe thorax or back pain
11	Blood pressure events	
11A	Hypertensive attack	Symptomatic or asymptomatic extreme high blood pressure
11B	Low blood pressure	Symptomatic or asymptomatic low blood pressure
12	Severe dyspnea	Hospitalization request due to extreme shortness of breath and decreased saturation
13	Lower extremity edema	Lower extremity edema that lasts more than a week and is responsive to medical treatment
14	Allergic reaction	Mild to moderate allergic reaction due to medication or food ingested
15	INR value disorder	
15A	Controlled	INR value 1.5–2 or 4–7
15B	Uncontrolled	Prone to bleeding or bleeding INR > 7

Abbreviations: COVID‐19, coronavirus disease 2019; ECG, electrocardiogram; INR, International Normalized Ratio (for prothrombin time).

A total of 5024 (6.3% per RME) repeat admissions to the hospital/doctor were prevented with the utilization of remote patient monitoring. In these cases, severe pain was observed in 2228 (2.8%) patients, hypertensive attack in 687 (0.9%) patients, atrial fibrillation in 198 patients (0.2%), and sinus tachycardia in 332 patients (0.4%)—all of which could be addressed or treated remotely with respect to the patients' expectations and/or requests (Table 3).

At the end of the follow‐up period, patients were asked to fill out a questionnaire and, overall, 96% (*n* = 2246) of patients reported that they were satisfied with the remote monitoring system. Additionally, 87% (*n* = 2035) of patients stated that they did not have difficulty in using the system and felt more comfortable psychologically, 98% (*n* = 2281) reported that the RMEs were effective, 82% (*n* = 1909) found that the information and warning module successfully addressed their specific condition, and 72% (*n* = 1684) stated that they wanted to extend the follow‐up period with this system.

## 4 DISCUSSION

Remote Patient Monitoring Systems began to spread all over the world even before the impact of the COVID‐19 pandemic.^16^, ^17^, ^18^, ^19^ However, with the COVID‐19 pandemic, these systems appear to have gained much more value. Throughout the world, the Health Ministries of numerous countries have provided incentives to construct and develop these systems.^20^, ^21^ Despite the pandemic process, the treatment, and surgeries of heart diseases must continue. In such a period, it is again the duty of health professionals to minimize unnecessary hospitalization and unnecessary travel. By utilization of available technology and integrating developments into health systems, significant benefits can be procured for patients, healthcare professionals, and healthcare policy‐makers. With the system we used in this study, the communication between the patients and our Telemedicine Center team was carried out in a professional manner, and potentially fatal complications were diagnosed early and treated in 118 (5%) patients.

Professional medical devices integrated into a system are required for communicating with patients and monitoring vital parameters. Thanks to these devices, the approximate severity and risks of patients can be determined. As a matter of fact, in 468 (20%) patients who developed conditions defined as complications, treatments were applied via various remote methods, including medication changes/adjustment, medical advice, video conferences, and exercises. During the 3‐year analysis period, 5024 hospital applications requested by patients were successfully addressed as a result of the examination of the Telemedicine team, and therefore, these unnecessary applications were prevented.

Telemedicine models, which have been used extensively until now, have generally focused on video conferencing or remote ECG monitoring.^22^, ^23^, ^24^ When it comes to evaluating a patient's state, all vital parameters (e.g., blood pressure, heart rate, oxygen saturation, body temperature, blood glucose, and live ECG), as well as postoperative needs, including medication reminder, automatic pathological data notification, message and video conference systems, are highly valuable. In addition, thanks to the daily information provided to patients by the system, a conscious patient group was formed, enabling adequate preparation for in‐person follow‐up studies. This effect is similar to that obtained through Clinical Rehabilitation programs.

It must be underlined that online examinations can never replace a professional in‐person examination at the clinic. However, these methods, when applied in a deliberate fashion, can prevent the feeling of loneliness in the face of disease and enable connections with health professionals when necessary. These factors appear to be very important for patients, as demonstrated by the percentage of patients reporting psychological relief by the use of this remote monitoring system (87%). For these reasons, online follow‐up systems have the potential to increase patient satisfaction and may provide a considerable decrease in hospital applications.

During the follow‐up period, the use of incorrect medication and incorrect dosage were prevented 628 and 422 times, respectively. Older patients often tend to prepare drugs that they need to use in advance which may cause incorrect dosage, but thanks to the medication reminder feature on the application of their smartphones, the dose and the name of the drug to be taken are described in an easy‐to‐understand and definite manner, which prevents erroneous medication use.

**Figure 3:**
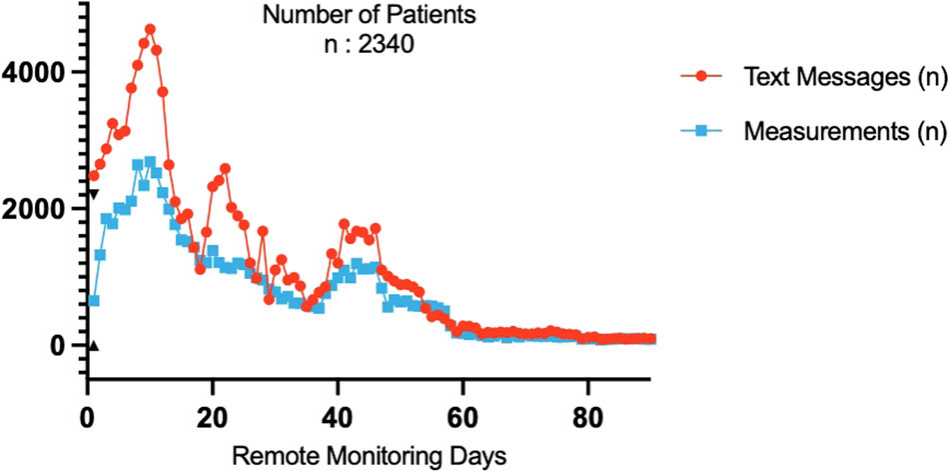
Text messages and measurements data sets are shown in an XY Graph in depending on time

The requests and measurements made by patients had a significant peak between 10 and 14 days, and then gradually declined. We think that this finding may be associated with patients feeling concerned and uncontrolled at home during the early period after discharged (Figure 3). Additionally, a second request peak was observed between 22 and 28 days, but the number of measurements was not increased in a similar manner, and the paramedical problems of the patients seemed to have a greater role. In parallel with the device measurements, we see that there is a considerable decrease in requests after the third and last peak. We attribute this third peak to mobilization and easing of movement restrictions after the first‐month in‐person follow‐up. The increase in pain during this period may have led to an increase in the number of requests and measurements performed. From the 52nd day on, the findings indicate that the majority of patients return to routine daily life.

In this study, a 6‐in‐1 device was used to measure all parameters of interest (Health Monitor DakikApp). This approach eliminates the need for several devices after discharge, thereby increasing compliance with the use of the remote monitoring system since all measurements were conducted from a single device. The second device (Holter ECG DakikApp) was used to record the heart rhythm of the patients, and they were treated remotely or by inviting for a check‐up in the presence of arrhythmia. Devices to be integrated into Remote Patient Monitoring systems can be further developed, and different devices can be used for different fields of medicine with the possible inclusion/replacement of other parameters with respect to patient‐specific needs.^25^, ^26^

The limitation of the study is that the outcomes of using remote monitoring could not be compared with a control group due to ethical considerations, and thus, all patients accepting to participate in the study received remote monitoring. We believe that a large number of patients and the experiences gained with this study can shed light on future studies and study goals in this field. Nonetheless, it may be valuable to re‐examined clinical outcomes with randomized prospective studies. However, it may prove to be very difficult to randomize follow‐up patient groups and draw reliable comparative data. Randomized studies in similar branches should be increased^27^, ^28^ and end‐points should be standardized to include all branches. By waiting for the results of randomized or matched‐group studies, it may be necessary to bring a systematic to the field of Telemedicine and even a branching out can be made in this field. It is also evident that, due to the novel nature of different monitoring systems and the relative immaturity of remote medicine, there is considerable confusion concerning the nature of remote monitoring systems. Universally accepted terms should be described in this field to identify which types/models of remote monitoring or remote patient care can be described as “Remote Patient Monitoring” or “Telemedicine.”

In light of the experience we have gained with the remote follow‐up of 2340 patients over a 3‐year period, we would like to emphasize that such concepts have a considerable impact on increasing the comfort of patients, and that they can increase the level of success thanks to the professional control of the postoperative follow‐up of primary cardiac surgery treatments. These effects are particularly important when the effects of COVID‐19 on patient care are considered.

## CONFLICT OF INTERESTS

The authors declare that there are no conflict of interests.
